# A Coin-Sized Oxygen Laser Sensor Based on Tunable Diode Laser Absorption Spectroscopy Combining a Toroidal Absorption Cell

**DOI:** 10.3390/s23198249

**Published:** 2023-10-05

**Authors:** Minxia Mao, Ting Gong, Kangjie Yuan, Lin Li, Guqing Guo, Xiaocong Sun, Yali Tian, Xuanbing Qiu, Christa Fittschen, Chuanliang Li

**Affiliations:** 1 Shanxi Engineering Research Center of Precision Measurement and Online Detection Equipment, Shanxi Center of Technology Innovation for Light Manipulations and Applications, School of Applied Science, Taiyuan University of Science and Technology, Taiyuan 030024, China; 2 CNRS, UMR 8522-PC2A—Physicochimie des Processus de Combustion et de l’Atmosphère, Université Lille, F-59000 Lille, France; christa.fittschen@univ-lille.fr

**Keywords:** TDLAS, VCSEL, oxygen concentration detection, toroidal absorption cell

## Abstract

Laser gas sensors with small volume and light weight are in high demand in the aerospace industry. To address this, a coin-sized oxygen (O_2_) sensor has been successfully developed based on a small toroidal absorption cell design. The absorption cell integrates a vertical-cavity surface-emitting laser (VCSEL) and photodetector into a compact unit, measuring 90 × 40 × 20 mm and weighing 75.16 g. Tunable diode laser absorption spectroscopy (TDLAS) is used to obtain the O_2_ spectral line at 763 nm. For further improving the sensitivity and robustness of the sensor, wavelength modulation spectroscopy (WMS) is utilized for the measurement. The obtained linear correlation coefficient is 0.9994. Based on Allen variance analysis, the sensor achieves an impressive minimum detection limit of 0.06% for oxygen concentration at an integration time of 318 s. The pressure-dependent relationship has been validated by accounting for the pressure factor in data processing. To affirm its efficacy, the laser spectrometer underwent continuous atmospheric O_2_ measurement for 24 h, showcasing its stability and robustness. This development introduces a continuous online laser spectral sensor with potential applications in manned spaceflight scenarios.

## 1. Introduction

Oxygen (O_2_) plays an indispensable role across diverse domains, encompassing human survival, industrial production, aerospace applications, and beyond [1,2]. Whether within the realm of rocket propulsion [3], space station functionality [4], or confined cabin environments [5], the real-time monitoring of oxygen provisioning and concentration emerges as an imperative. It aims to abate the potential hazards stemming from anomalies in oxygen concentration, which could precipitate explosions or life-threatening occurrences. However, taking into account factors such as optimized spatial resource allocation and research and development funds, the demand for oxygen sensors characterized by traits of being lightweight, miniaturized, and seamlessly integrated is progressively growing, warranting in-depth research.

Tunable diode laser absorption spectroscopy (TDLAS) has been successfully employed in detecting gases due to its advantage of high precision, high selectivity, high sensitivity and noncontact measurement [6,7,8]. To improve the detection sensitivity, the utilization of multipass cells (MPCs) with enhanced optical path length has become customary. Various MPCs have been developed, including White absorption cells [9,10], Herriott absorption cells [11,12,13], Chernin absorption cells [14] and toroidal absorption cells [15,16].

Among these MPC variations, the toroidal absorption cell distinguishes itself with its singular torus configuration, furnishing a stable and robust structural framework. The optical path length can be conveniently and flexibly adjusted by manipulating the incidence angle. In practical applications, independent structures facilitate the use of toroidal absorption cells in large-scale measurement environments, such as the isotope of methane gas [15], carbon dioxide [17,18] or nitrogen dioxide [19]. However, a common limitation of existing sensors is their incorporation of external optical paths along with lens assemblies and reflectors, which weakens their anti-vibration effect [20]. Consequently, there is a growing trend towards integrated designs to mitigate vibration effects, reduce size, and enhance portability.

TO-packaged vertical-cavity surface-emitting lasers (VCSELs) present a range of benefits, including superior beam quality, minimal divergence angles, low power consumption, elevated efficiency, compact dimensions, prolonged operational longevity, and economically viability [21,22,23]. Furthermore, owing to their intricate design, VCSELs can be seamlessly integrated within the toroidal absorption cell, thus serving the broader objective of comprehensive design endeavors.

In this research, a coin-sized TDLAS sensor was developed based on VCSEL combining with a miniaturized toroidal absorption cell, with a size of 90 × 40 × 20 mm and a total weight of 75.16 g. The sensor’s analytical performance was rigorously evaluated through measurements of atmospheric oxygen (O_2_) levels. The wavelength modulation spectroscopy (WMS) and the Kalman filter were employed for improving the sensitivity. Allan deviation was utilized to analyze the stability of the sensor. To affirm the sensor’s practical utility, we demonstrated continuous atmospheric O_2_ monitoring over a full day, showcasing its applicability for prolonged and real-time detection.

## 2. Theory

TDLAS technology utilizes a tunable semiconductor laser to emit light at a specific frequency (wavelength) into the gas to be measured, thus facilitating the determination of various gas parameters. The entire process follows the Beer–Lambert Law Equation (1), and the degree of laser energy absorption depends on the gas concentration. The gas concentration to be measured is calculated as

(1) $$\begin{array}{cc} I(t) & =I_{0}(t)\exp[-N\sigma (\nu )L]=I_{0}(t)\exp[-\alpha (\nu )L]\\ & =I_{0}(t)\exp[-P_{tot}xS^{*}L\varphi (\nu )] \end{array},$$

where $I_{0}(t)$ is the laser intensity without the absorbing medium, I(t) is the transmitted intensity, *L* is the optical length, *N* (in molecule/cm^3^) is the number of absorbing molecules per cubic centimeter, σ(ν) (in cm^2^/molecule) is the absorption cross-section, and α(ν) is the absorption coefficient. $P_{tot}$ is the total pressure in the cell, x is the mole fraction of the absorbing species, and φ(ν) is the line shape function, and its integration is normalized to 1. The line strength $S^{*}$ (in cm^−2^/atm) is calculated from the value of S (in cm^−1^/(molecule·cm^−2^)) obtained from the HITRAN database in the following equation:

(2) $$S^{*}=SN_{0}\frac{T_{0}}{T}=7.34\times 10^{21}\frac{S}{T},$$

where $N_{0}$ = 2.6868 ×10^19^ molecule/cm^3^ is the number density of an ideal gas at $T_{0}$ = 273.15 K and $P_{0}$ = 760 Torr.

The integrated absorbance area $A_{I}$ (in cm^−1^) under the absorption line profile can be approximated as

(3) $$\begin{array}{cc} A_{I} & =\int \ln(I_{0}/I)d\nu =NL\int \sigma (\nu )d\nu \\ & =P_{tot}xS^{*}L\int \varphi (\nu )d\nu =P_{tot}xS^{*}L=NSL \end{array},$$

The total number density $N_{tot}$ (in molecule/cm^3^) in the cell can be determined with

(4) $$N_{tot}=\frac{P_{tot}T_{0}}{P_{0}T}N_{0},$$

where *T* is the temperature in the cell. Therefore, $N/N_{tot}$, concentration ratio of the absorbing molecules, can be obtained based on Equations (3) and (4).

Wavelength modulation technology (WMS) represents a significant advancement in enhancing the precision of spectral measurements. To mitigate noise during spectral measurement, it becomes imperative to shift the output signal from a low frequency range to a higher frequency range. A widely employed technique for accomplishing this involves the addition of a high-frequency sine wave signal to the injection current of the laser. Subsequently, the spectral signal recorded by the detector is subjected to demodulation via a phase-locked amplifier. This strategic approach effectively curbs noise stemming from both the laser and the inherent characteristics of the detector. Consequently, this methodology contributes to an exponential enhancement in the signal-to-noise ratio. For WMS measurement, the diode laser injection current is modulated with angular frequency ω=2πf. Thus, the modulated output laser wavelength and intensity are

(5) $$\begin{array}{l} \nu \left(t\right)=\bar{\nu }+a\cos\left(\omega t\right)\\ I_{0}={\bar{I}}_{0}\left[1+i_{1}\cos\left(\omega t+\psi_{1}\right)+i_{2}\cos\left(2\omega t+\psi_{2}\right)\right] \end{array},$$

where $\bar{\nu }$ is the center laser frequency and a is the modulation depth. $I_{0}$ is the peak-peak value of the modulation signal detected via the photodetector. The parameters such as $i_{1}$, $i_{2}$, $\psi_{1}$ and $\psi_{2}$ describe the characteristics of the laser. For a more convenient and accurate description of signal, the second-order Taylor series of laser transmission τ is obtained using

(6) $$\begin{array}{cc} \tau \left(t\right) & =\frac{I}{I_{0}}=\exp[-P_{tot}xS^{*}L\varphi (\nu )]\\ & \approx 1-P_{tot}xS^{*}L\sum_{k=0}^{\infty }H_{k}\cos\left(k\times 2\pi ft\right)+\frac{P^{2}{}_{tot}x^{2}S^{*}{}^{2}L^{2}{\left[\sum_{k=0}^{\infty }H_{k}\cos\left(k\times 2\pi ft\right)\right]}^{2}}{2} \end{array},$$

The $H_{k}$ coefficients are described as

(7) $$H_{0}=\frac{1}{2\pi }\int_{-\pi }^{\pi }\varphi (\upsilon )d\theta ,$$

(8) $$H_{k}=\frac{1}{\pi }\int_{-\pi }^{\pi }\varphi (\upsilon )\cos(k\theta )d\theta ,$$

In WMS technology, lock-in amplifiers are used to obtain high-order harmonic signals based on orthogonal demodulation schemes. The amplitude of odd harmonics is zero at the center of the absorption spectrum, while even harmonics have a significantly strong amplitude and the signal amplitude decreases with the increase in even harmonics. Therefore, using second harmonic signals (2*f*) to invert the concentration of gases is an excellent choice. In spectrum measurements, the amplitude of 2*f* is approximately represented as

(9) $$S_{2f}\approx -\frac{1}{2}G{\bar{I}}_{0}P_{tot}xS^{*}LH_{2}$$

where *G* is the optical-electrical gain of the detection system.

## 3. Experiments

**Figure 1:**
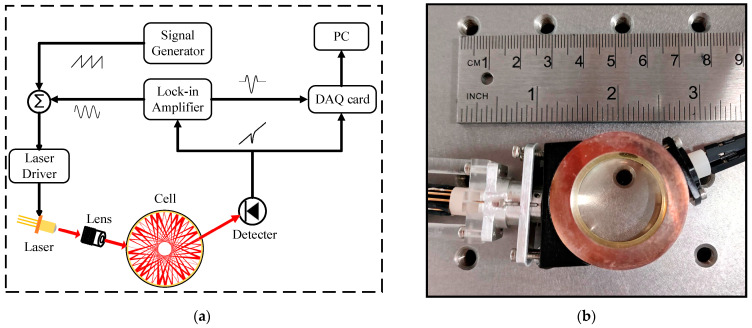
(**a**) Schematic diagram of the experimental setup for O_2_ detection; (**b**) photograph of the real instrument.

The experimental setup is depicted in Figure 1a. The VCSEL’s central wavelength, approximately 763 nm, is systematically scanned utilizing a laser driver control system (ILX Lightwave (USA), LDC-3724C). To converge the laser at the cell’s center, an adjustable focal length lens is employed, with a spot size of 37 μm, effectively suppressing optical interference fringes and optimizing cell utilization. Within the cell, the laser undergoes multiple reflections, engaging in interactions with the gas sample. This interaction pattern generates a distinctive polygonal star spot configuration with an effective optical path length of 1 m. Subsequently, the transmitted laser light is captured with a photodetector and demodulated at the second harmonic frequency through a lock-in amplifier. The resulting demodulated signal is captured via a data acquisition card (PCI-6335) and analyzed using a personal computer. The performance of the sensor is characterized for a series of O_2_ concentration measurements. The sensor is placed in a sealed vacuum hood for concentration calibration measurement. The sample gas for concentration measurements were supplied by diluting a calibrated gas mixture of 100%, 20% and 10% O_2_ with pure nitrogen (N_2_).

The actual instrument configuration is depicted in Figure 1b. The TDLAS sensor, designed to match the dimensions of a coin, integrates a photodetector component, a miniature cell, and a laser component. This amalgamation is achieved through a mechanical framework that not only ensures structural coherence but also facilitates optical path adjustments. The cell is precision-machined from a material that holds high polish and corrosion resistance. Its interior surface is coated with a silver film, possessing a remarkable reflectivity of 98% in near-infrared spectral ranges, thereby curtailing optical power losses. To prevent contamination of the inner wall of the toroidal absorption cell during the measurement process, we applied transparent protective films both above and below it. The cell’s dimensions stand at a diameter of 38 mm and a height of 18 mm. The physical dimensions of the sensor are minimized to 90 × 40 × 20 mm, with an overall mass of merely 75.16 g. This miniaturization renders the sensor exceptionally portable, characterized by its diminutive size, lightweight construction, and easy transportability.

## 4. Discussion

**Figure 2:**
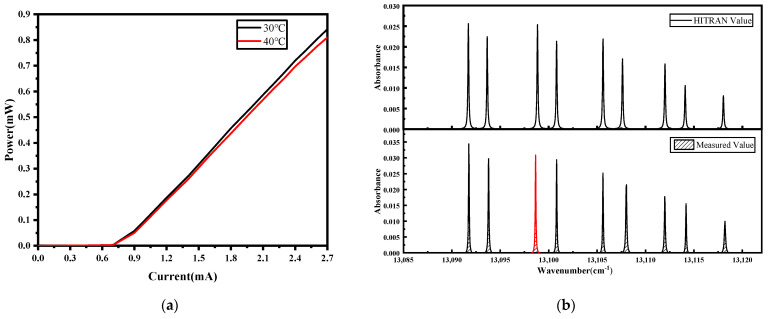
(**a**) The relationship between the output power and the current of VCSEL; (**b**) the absorption spectrum line of the HITRAN database and measured absorption spectrum line of 20% O_2_ at 1 atm.

The relationship between the output power and the current of VCSEL is shown in Figure 2a. Conforming to constraints imposed by current thresholds and maximum values, a scan range of 1.62 to 2.41 mA is established. By modulating the laser’s temperature, nine absorption peaks were acquired, covering the span of the light emission spectrum, as illustrated in Figure 2b. These spectra exhibited a high level of congruence with data provided by the HITRAN database (as evidenced in Figure 2b). After deliberating on the imperative factors of stable operational conditions and absorption intensity, the third oxygen absorption spectral line is selected for further analysis (indicated by the red spectral line in the figure).

**Figure 3:**
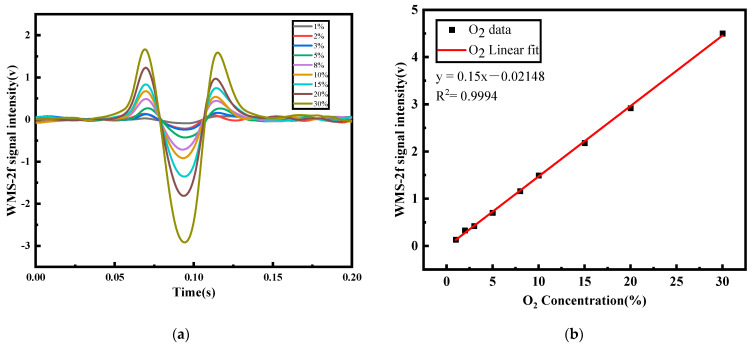
(**a**) WMS-2*f* signal for 1–30% oxygen concentrations; (**b**) linear relationship between the WMS-2*f* signal intensity of O_2_ and the real concentrations.

The sensor performance was investigated by measuring a series of O_2_ mixtures of known concentrations. The second harmonic signal obtained through wavelength modulation spectroscopy (WMS-2*f*) for various oxygen concentration at atmospheric pressure is presented in Figure 3a. The relationship between intensity of WMS-2*f* signals and O_2_ concentration is shown in Figure 3b. A good linear relationship can be observed, with a correlation coefficient R^2^ of 0.9994. The O_2_ concentration can be inferred using the formula *C* = (Peak (WMS-2*f*) − 0.02148)/0.15.

**Figure 4:**
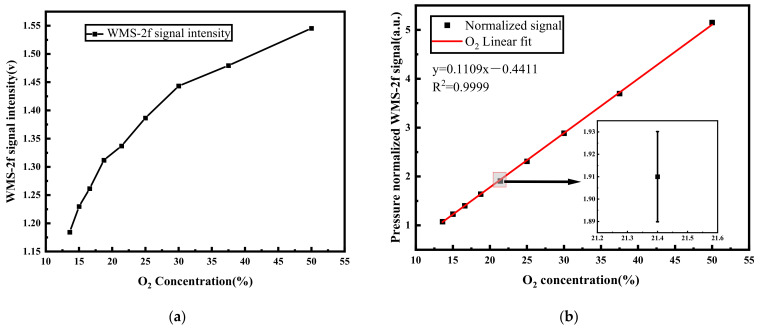
Fifty percent of O_2_ diluted with nitrogen gas at 0.3 atm. (**a**) The real concentrations versus the WMS-2*f* signal amplitude; (**b**) the pressure-dependent relationship between the signal intensity and the real O_2_ concentrations. The insertion diagram is an enlargement of the error bar.

In the presence of varying pressure conditions, molecules demonstrate distinct collision frequencies and distributions of molecular thermal motion velocities, subsequently influencing the characteristics of their spectral line profiles. This alteration can have an impact on the accuracy of oxygen concentration measurements. Therefore, it is imperative to account for the influence of pressure on the 2*f* signal. During the experimental process, 0.3 atm 50% O_2_ was first filled into the cell, followed by the gradual infusion of pure N_2_ to induce oxygen concentration dilution, concurrently leading to changes in pressure inside the cell. Figure 4a illustrates the relationship between WMS-2*f* signal intensity and oxygen concentration, showing a rapid decline in WMS-2*f* signal intensity as oxygen concentration undergoes dilution. The interrelation among pressure, concentration, and the 2*f* signal can be deduced from Equation (9). To mitigate the influence of pressure on the results, we normalized the pressure values by dividing the 2*f* signal at a specific concentration by the pressure value, as shown in Figure 4b. Linear fitting was employed, and an R^2^ value of 0.9999 revealed the strong linear correlation. Consequently, when the ambient pressure is known, oxygen concentration measurement can be directly inferred through this linear relationship.

**Figure 5:**
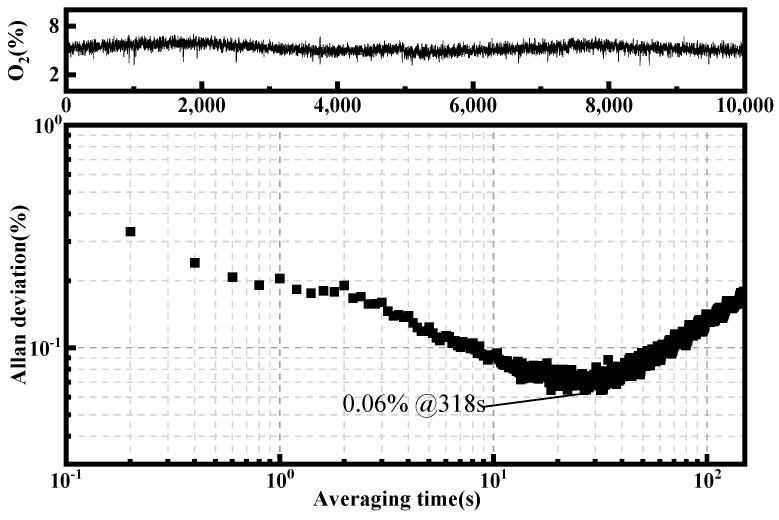
Row data of individual concentration measurements during more than 3 h (**top**) and Allan variance as a function of integration time (**bottom**).

To affirm the sustained stability of the O_2_ sensor, Allan deviation analysis was employed for an in-depth evaluation of its long-term performance, as shown in Figure 5. It indicates that the optimum integration time is 318 s, and the corresponding detection limit is 0.06%.

We compared our home-made oxygen sensor with the work of others, as shown in Table 1. Among them, Gurneesh et al. [13] and Chang et al. [20] achieved detection limits of 0.1% and 0.022% at output power of 12 mW and 6 mW, and optical path of 4.3 m and 3.3 m, respectively, by combining DFB lasers with Herriott cell. Although the detection limit obtained by Chang et al. is three times lower than ours, its optical path and output power are much greater than ours. The laser used in Gao et al. [22] is the same as that used in this paper, both being VCSEL. Under the same output power and optical path conditions, the detection sensitivity of our sensor has been improved by an order of magnitude. These data fully confirm the advantages of our designed sensor, which can achieve impressive detection sensitivity in small volume and low power.

**Figure 6:**
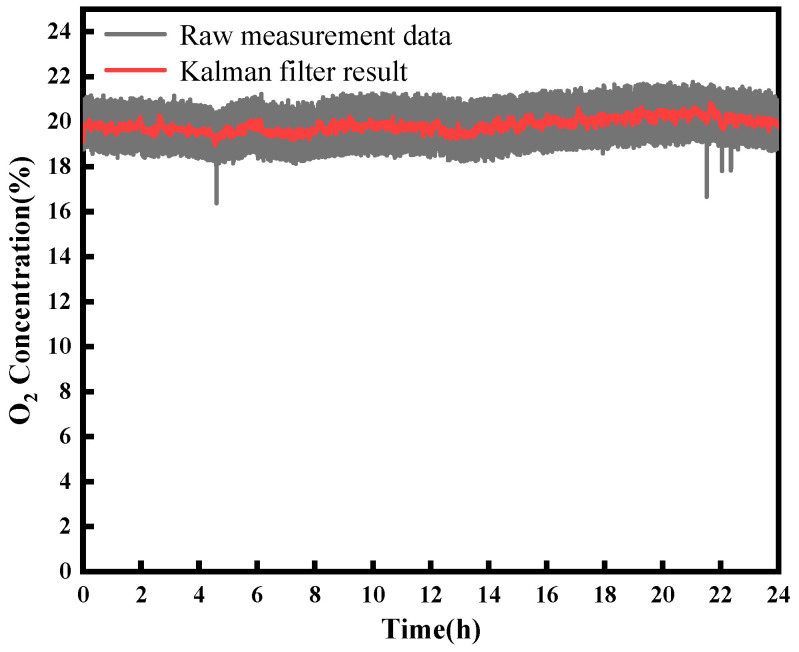
A Kalman filter was used to improve the detection precision (red lines).

Continuous monitoring of atmospheric oxygen was conducted over a 24 h period utilizing sensors positioned across the campus of Taiyuan University of Science and Technology, as illustrated in Figure 6. The raw measurement data, depicted by the gray line, exhibit a standard deviation of 0.83%. By employing a Kalman filter, noise was reduced, resulting in an enhancement of measurement accuracy. The application of the filter reduces the standard deviation to 0.29%, marking an impressive 2.86 times enhancement in system accuracy as illustrated by the red line in Figure 6. According to the relationship between oxygen content in the atmosphere and altitude, the oxygen content in the atmosphere of Taiyuan City is about 19.67%. Notably, the experimental results exhibited remarkable consistency with these expectations, thereby confirming the precision and reliability inherent to our sensor system.

## 5. Conclusions

A compact TDLAS sensor, employing a minute toroidal absorption cell combined with a low-power VCSEL, designed to match the dimensions of a coin was assembled and used to accurately measure O_2_ concentrations. Employing Allan variance analysis, the sensor system achieves an impressive detection limit of 0.06%, showcasing the feasibility of the TDLAS system. The applicability of the system’s performance is substantiated by a continuous 24 h monitoring of ambient air O_2_ concentrations. Furthermore, the Kalman filter was tested to improve the gas concentration accuracy, leading to an approximately 2.86 times enhancement. In order to take into account the influence of varying environmental pressure, we have normalized the pressure values. This methodology allows for favorable concentration inversion values and reduced measurement errors under varying pressure conditions. The developed TDLAS system establishes itself as a potential measuring tool for monitoring oxygen concentrations in aerospace applications.

## Figures and Tables

**Table 1:** Comparison table of performance indicators of oxygen sensors.

Power (mw)	Length (m)	Detection Limit (%)	Refs.
12	4.3	0.1	[13]
6	3.3	0.022	[20]
0.6	1	0.1	[22]
0.6	1	0.06	Our work

## Data Availability

The raw data supporting the conclusions of this article will be made available by the authors, without undue reservation.
